# Coordinating Qualitative Predictor Variables in an Applied Linear Model: Analysis and Application for Applied Sciences

**DOI:** 10.7759/cureus.59151

**Published:** 2024-04-27

**Authors:** Wan Muhamad Amir W Ahmad, Faraz Ahmed, Mohamad N Adnan

**Affiliations:** 1 Dental Sciences, Universiti Sains Malaysia, Kelantan, MYS; 2 Biostatistics, Universiti Sains Malaysia, Kota Bharu, MYS; 3 Dental Sciences, Universiti Sains Malaysia, Kota Bharu, MYS

**Keywords:** bootstrapping, hybrid methodology, mlffnn, fuzzy regression, qualitative predictors, linear regression

## Abstract

Background

In applied sciences, statistical models are pivotal for uncovering relationships in complex datasets. The applied linear model establishes associative links between variables. While qualitative predictors are essential, their integration into linear models poses challenges. The dummy variable approach transforms qualitative variables into binary ones for regression analysis. Multilayer Feedforward Neural Networks (MLFFNN) offer validation of regression models, and fuzzy regression offers alternative methods to address the ambiguity of qualitative predictors. This study aims to enhance the integration of qualitative predictors in applied linear models through statistical methodologies.

Material and methods

This study design involves the transformation of qualitative predictors into dummy variables, the bootstrapping technique to improve the parameter estimates, the Multilayer Feedforward Neural Network, and fuzzy regression. This study uses the programming language R as an analysis tool.

Results

The multiple linear regression model demonstrates precision and a significant fit (p<0.05), with an R-squared value of 0.95 and mean square error (MSE) of 9.97. Comparing actual and predicted values, fuzzy regression exhibits superior predictability over linear regression. The MLFFNN yields a reduced MSE net of 0.362, indicating enhanced prediction precision for derived models.

Conclusion

This study presents a precise methodology for integrating qualitative variables into linear regression, supported by the combination of specific statistical methodologies to enhance predictive modeling. By integrating fuzzy linear regression, MLFF neural networks, and bootstrapping, the proposed technique emerges as the most effective approach for modeling and prediction. These findings underscore the efficacy of this method in seamlessly integrating qualitative variables into linear models, ultimately enhancing accuracy and prediction capabilities.

## Introduction

In the field of applied sciences, the application of statistical models plays a vital role in revealing the diverse relationships between variables, trends, and insights from complex data sets. Likewise, the applied linear model is a statistical technique used to establish a causational or associative relationship between two or more variables [1]. Its purpose is to predict the response of one variable based on the conditions of the other variables [1]. For instance, the multiple linear regression model is a statistical method for modeling and investigating the influence of explanatory variables on a response variable [2]. However, when dealing with qualitative predictors, such as categorical variables, a conventional linear model requires careful consideration and specific techniques for accurate prediction [3]. In multiple linear regression (MLR), qualitative variables, in addition to scale variables, can have an impact on the results [4,5]. Many researchers have used qualitative predictors in their prediction models as an important variable [1,6]. However, due to their qualitative nature, these variables cannot be incorporated directly into the linear regression. Therefore, the integration of qualitative predictors into the applied linear models is an intricate process that involves a detailed understanding of statistical methods and their practical impacts [7]. Qualitative variables can be brought within the regression scope using a very common method known as the "dummy variable approach" [8]. The dummy variable approach is a statistical process where qualitative variables are transformed into binary variables, such as zero (0) and one (1), which indicate the absence or presence of a variable of interest, respectively [9]. The application of qualitative predictor variables in a regression analysis may increase model precision and outcome quality. A review of the current literature revealed that the handling of qualitative predictors while fitting a regression model is an important problem that is currently inadequately discussed [10].

Qualitative variables introduce a level of fuzziness or ambiguity due to their non-numeric nature, making their integration into traditional linear regression models challenging [11]. Fuzzy linear regression addresses this issue by allowing for the modeling of relationships between qualitative variables and quantitative outcomes in a more flexible and nuanced manner. Tanaka et al. (1982) developed a fuzzy regression model with fuzzy responses, fuzzy parameters, and non-crisp response data [12]. Kovac et al. (2013) employed both a fuzzy logic model and a linear regression model to predict surface roughness as a machining parameter and concluded that the fuzzy regression model outperformed the linear regression model in terms of effectiveness [13]. In the context of validating derived models, Multilayer Feedforward Neural Networks (MLFFNN) serve as a powerful tool [14]. MLFFNN is used as a validation approach for regression models in this study. This method ensured that the independent variables used in the regression analysis were valid. Ahmad et al. developed a model to predict triglycerides' relationship to waist circumference, high-density lipoprotein (HDL), and hypertension. Small MLFFNN root mean square error (MSE net) validated the regression model [15].

The significance of this research stems from the widespread presence of qualitative variables in applied sciences. Unlike quantitative predictors, qualitative predictors introduce unique challenges due to their discrete and non-numeric nature. Consequently, a robust methodology for incorporating these variables is imperative to enhance the accuracy and interpretability of linear models in diverse scientific applications. Therefore, the objective of the current study is to coordinate qualitative predictors in applied linear models by integrating specific statistical methodologies, aiming to develop prediction models that are both more accurate and precise compared to traditional methods while also seeking to find a more efficient way to estimate model parameters and enhance interpretability for qualitative data. 

## Materials and methods

Study design

**Figure 1:**
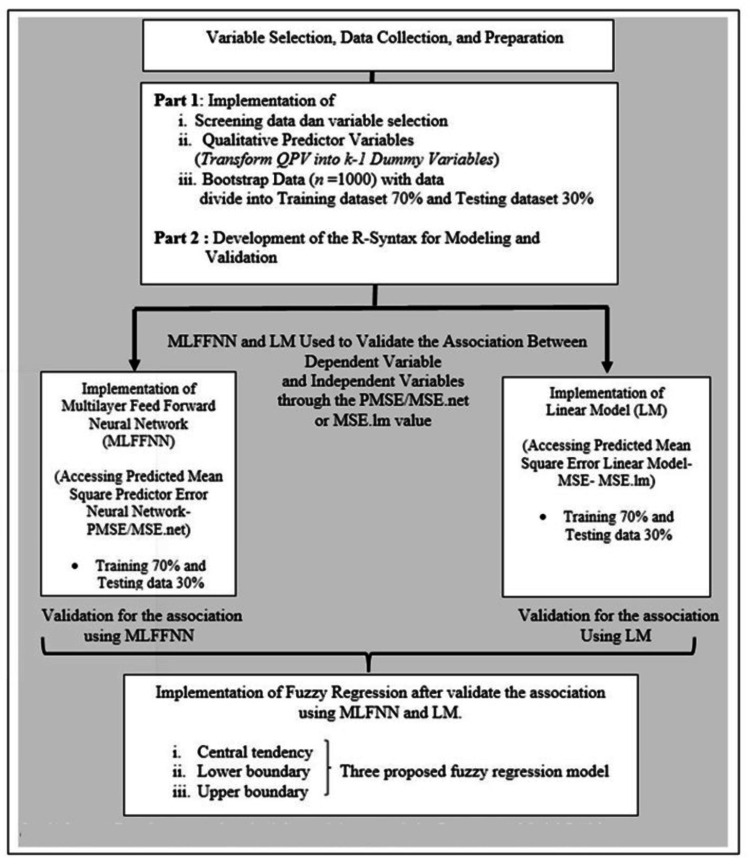
The conceptual framework of the proposed methodology

This study utilizes a computational biostatistical study design. This study design involves the screening of data and variable selection, the transformation of qualitative predictors into dummy variables, the bootstrapping technique to improve the parameter estimates, Multilayer Feedforward Neural Network (MLFFNN), and fuzzy regression. R-software (R Foundation, Vienna, Austria) was employed as a research tool in this research. Figure 1 shows the conceptual framework of the development method.

The conceptual framework (Figure 1) outlines a systematic approach to addressing complex problems by coordinating qualitative predictors within applied linear models. The process starts with variable selection, emphasizing clinically relevant variables from a secondary dataset in health science. The dataset includes both quantitative and qualitative variables. Qualitative variables are transformed for compatibility using a dummy variable approach, followed by bootstrapping to enhance data precision. The dataset is split into a 70:30 ratio for multiple linear regression (MLR) model development and validation. MLR models incorporate both types of predictors, and variable associations are validated using mean square error (MSE). Additional models, including multilayer feedforward neural networks and fuzzy linear regression, are developed post-validation to explore predictive capabilities. Three fuzzy regression models are proposed to address uncertainty from qualitative predictors. Overall, the framework offers a structured method to develop robust prediction models tailored to health science.

Computational biostatistics modeling

R-software is used for data analysis, and R-syntax in this study is a combination of the specific statistical techniques, which include bootstrapping, multiple linear regression, a multilayer feedforward neural network for validation, and fuzzy linear regression syntaxes.

Data sources

The dataset employed for this study originates from the book "Biostatistics" authored by Daniel and Cross in 2018 [16] and serves as the basis for evaluating the developed methodology. Table 1 displays the main characteristics of the data. The dataset collected data on treatment effectiveness, patient age, and specific treatments for severe depression. The dependent variable was treatment effectiveness, while age and treatment type were independent variables. The qualitative variable of treatment type included three distinct treatments. Detailed information regarding the administration of drugs was not revealed to maintain confidentiality. Additionally, for the sake of simplicity, the treatments were referred to as A, B, and C.

**Table 1:** Effectiveness of treatment among the different types of treatment Source: Daniel and Cross [16]

Effectiveness of treatment	Age (years)	Type of treatment
56	21	A
41	23	B
40	30	C
28	19	C
55	28	B
...	....	....
71	63	A
56	21	A

Bootstrapping (case resampling technique)

Bootstrapping is a fundamental statistical interference method that includes resampling a sample many times to generate a sample distribution for statistical purposes. Efron proposed the bootstrapping methodology, a promising alternative computerized technique, in 1979 [17]. The bootstrap does not produce a new sample; rather, it replaces the existing data values for the population and takes simulated samples from within the sample. This is achieved by taking a sample (via replacement) and building a larger sample "composed of case resampling", also known as bootstrap samples [18]. In general, bootstrapping improves the predictability and accuracy of the regression model.

Multiple linear regression with qualitative predictor

Multiple linear regression is a statistical method used to model and investigate the impact of explanatory variables on a response variable. The general MLR model for data and variables presented in Table 1 can be written as follows:

Equation 1: $Y = \beta _{o} + \beta _{1}X_{1} + \beta _{2}X_{2}$

Where Y refers to the effectiveness of the treatment, X_1_ refers to the age of years, and X_2 _refers to the type of treatment.

In this dataset, the variable treatment type (X_2_) has three additional categories, and because the nature of qualitative predictors cannot be directly incorporated into the linear regression, these qualitative predictor variables must be converted into an α-1 dummy variable. This conversion of variables into numeric form is known as a dummy variable approach. Dummy variables typically have only two values, "0" and "1", and if qualitative variables have the α category, α-1 dummy variables must be included in the model. Therefore, following the dummy variable approach, two indicator variables are needed. The transformation of the type of treatment (X_2_) is given by:

$Treatment A = \begin{Bmatrix} 1 & if Treatment A\\ 0& Otherwise \end{Bmatrix}$

$Treatment B = \begin{Bmatrix} 1 & if Treatment B\\ 0& Otherwise \end{Bmatrix}$

A first-order regression model is (equation 2): effectiveness = β0 + β1 Age + β2 Treatment A + β3 Treatment B

For this model, the data input for the X_2_ variables would be as follows (Table 2):

**Table 2:** Data input for categorical variable (X2)

Treatment	X_2_	Code assigned for predictor	
Treatment A	X_i2_	1	0
Treatment B	X_i2_	0	1
Treatment C	X_i2_	0	0

The response function for regression equation 2 with the indicator variables is given by:

Equation 3: E{Effectiveness} = β0 + β1 Age + β2 Treatment A + β3Treatment B

To understand the meaning of the regression equation 3 coefficient, consider first what response function 2 becomes if Treatment A = 0 and Treatment B = 0:

E{Effectiveness} = β0 + β1 Age

For Treatment A, replace Treatment A = 1 and Treatment B = 0, and the response function can be written as:

E{Effectiveness} = (β0 + β2)+ β1 Age

For Treatment B, replace Treatment A = 0 and Treatment B = 1, and the response function can be written as:

E{Effectiveness}= (β0 + β3) + β1 Age

Fuzzy linear regression (FLR)

Fuzzy regression enables the modeling and analysis of data that is uncertain, imprecise, or ambiguous, in contrast to standard linear regression, which deals with crisp or precise information [19]. Fuzzy regression is a method used to assess the functional relationship between the dependent and independent variables in a situation where there is uncertainty or ambiguity [15]. In contrast to traditional linear regression, where the parameters are treated as random variables with probability distribution functions, the coefficients in fuzzy regression are subject to possibility theory [20]. The general fuzzy linear regression model for n independent variables can be written as

Equation 4: $\widetilde{Y} = \widetilde{A_{o}} + \widetilde{A_{_{1}}}x_{1}+...+\widetilde{A_{n}}x_{n}$ or $\widetilde{Y_{j}} = \widetilde{A_{0}}+ \sum_{j=1}^{n} \widetilde{A_{j}}x_{j}$

**Figure 2:**
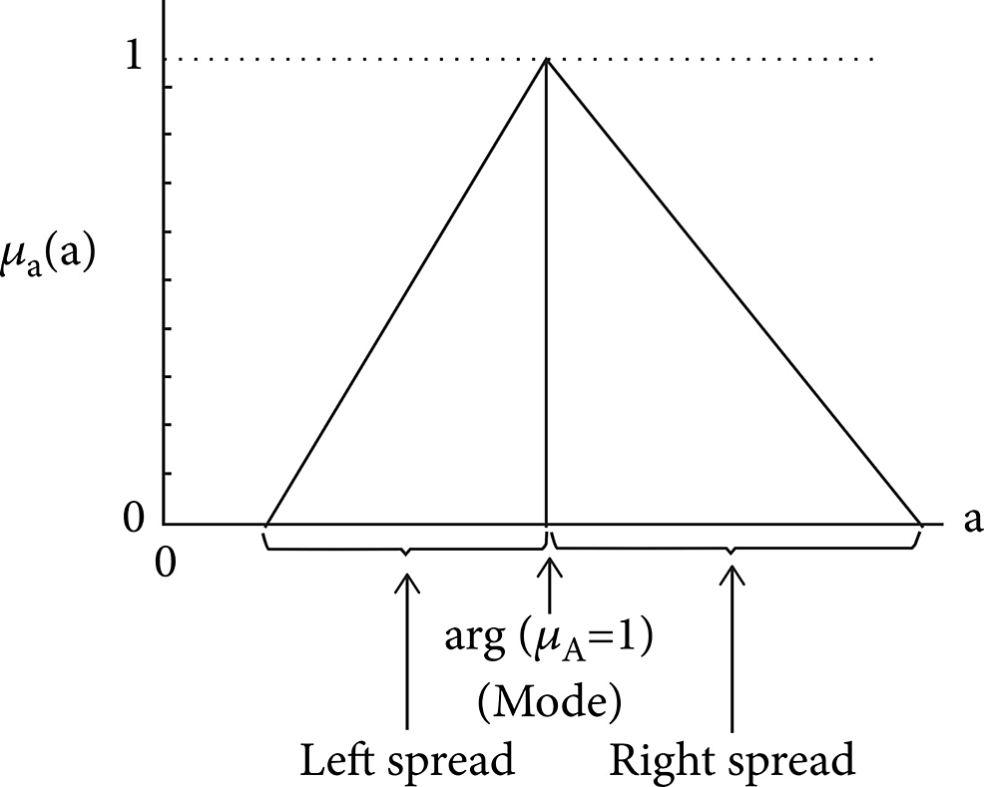
Fuzzy coefficient Source: Ahmad et al. [15]; Permission obtained

Where $\widetilde{Y}$ = Fuzzy output, x_j_ = [x_1_, x_2_, x_3_,...,x_n_] is the dimension non-fuzzy input vector, and $\widetilde{A_{j}}$ [*j*=1,2,3,...,n] is a fuzzy coefficient, which is displayed in Figure 2.

**Figure 3:**
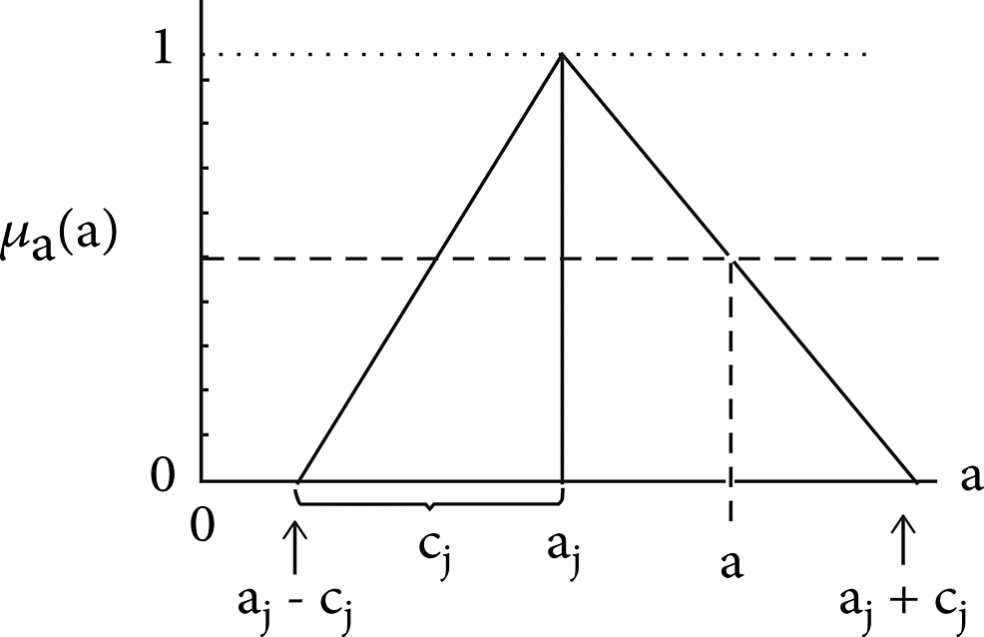
Fuzzy parameters Source: Ahmad et al. [15]; Permission obtained

Let's assume these parameters can be defined using a symmetric triangular membership function, which is presented in Figure 3.

Defining the fuzzy parameters from Figure 3,

Equation 5: $\widetilde{A_{j}}$ = { a_j_, c_j_} = {$\widetilde{A_{j}}$: a_j_ - c_j_ ≤ $\widetilde{A_{j}}$ ≤ a_j_ + c_j_ } and *j* = 0,1,2,...,n

Where a_j_ is center, and c_j_ represents vagueness associated.

By limiting our analysis to situations where just the coefficients are uncertain, we may express the following equation:

Equation 6: $\widetilde{Y_{j}}=\widetilde{A_{o}} + \sum_{n}^{j-1} \widetilde{A_{j}}x_{j}=(a_{o},c_{o}) + \sum_{n}^{j-1} (a_{j},c_{j}) x_{j}$

Multilayer Feed Forward Neural Network (MLFFNN)

A multilayer feedforward neural network was applied, which is widely used in the field of health-related studies. MLFFNN ensures the integrity of the independent variables utilized in regression analysis. Within a neural network, there are two primary steps: training and testing. Split the dataset into two segments: allocate the initial portion, typically constituting over 70% of the data, for training the network, and designate the remaining 30% for testing its performance. The MSE was measured during network testing, and a small error helped validate the regression model. A multilayer feedforward neural network is an interconnected artificial neural network with multiple hidden layers that have neurons with the weight associated with them and compute the results using an activation function [21]. MLFFNN is a type of neural network in which signals flow unidirectionally from input to output units. Since this study has a single dependent variable, the output node is unique in the investigation sample. The construction of general MLFFNN is based on the following equation: N input nodes, H hidden nodes, and one dependent output variable.

$\widehat{Y} = g_{i} \sum_{j=1}^{2} n_{j} + E_{i}$

Where g is the activation function and Ei is the bias for the output node. The value of Y is given as:

$\widehat{Y} = g_{2} \sum_{j=1}^{2} n_{j} + E_{2}$

**Figure 4:**
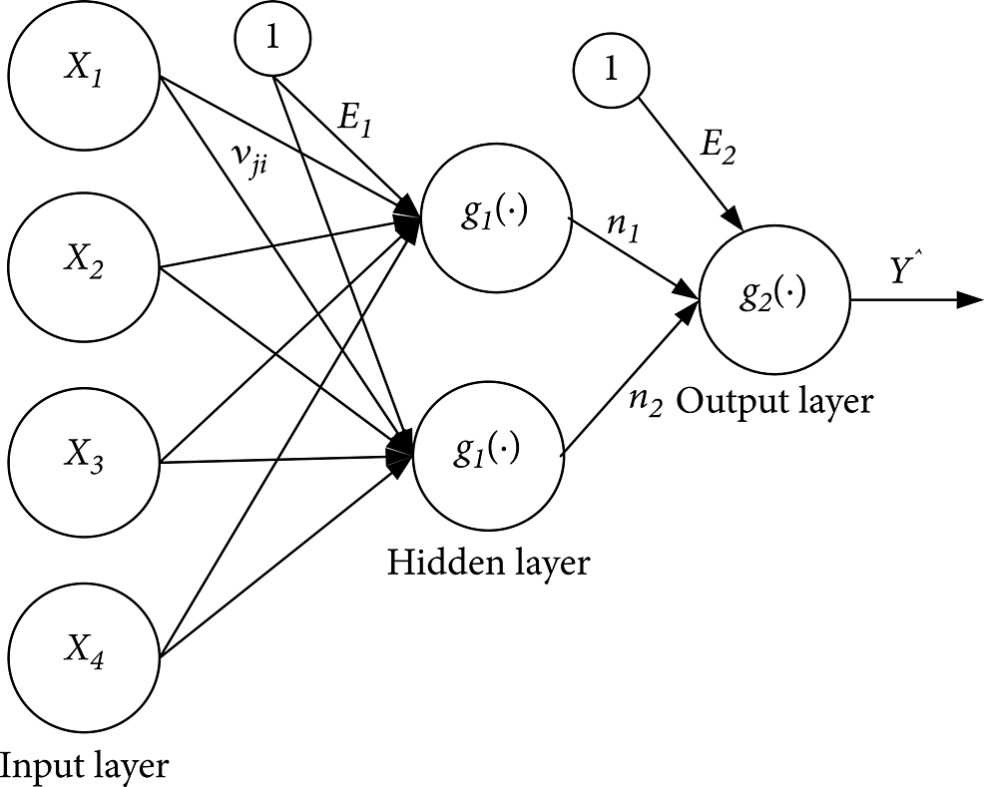
Architectural illustration of MLFFNN with input, hidden, and output layers Source: Ahmad et al. [15]; Permission obtained MLFFNN - Multilayer Feedforward Neural Networks

Where E2 is the bias for the output node and g is the activation function. The value of hidden node n_j_ is given as follows:  $n_{j} = g_{i} \sum_{j=1}^{2} v_{ij} + E_{1}$ where E_1_ is the bias of the output node j, and j = 1,2, and g is the activation function. The v_ij_ is the output weight from input node i to hidden node j. and x_i_ are the independent variables. Figure 4 is the general illustration of MLFFNN architecture.

The developed R syntax

The entire methodology is written in the R programming language.

Input =("

Measure age treatA treatB

56 21 1 0

41 23 0 1

. . . .

70 67 1 0

71 63 0 0

")

data = read.table(textConnection(Input),header=TRUE)

#/Performing Bootstrap for 10000

mydata <- rbind.data.frame(data, stringsAsFactors = FALSE)

iboot <- sample(1:nrow(mydata),size=10000, replace = TRUE)

bootdata <- mydata[iboot,]

#/Randomly split the data into 70:30

index = sample(1:nrow(bootdata),round(0.70*nrow(bootdata)))

train_data <- as.data.frame(bootdata[index,])

test_data <- as.data.frame(bootdata[-index,])

# /Fit a Linear Regression Model and compute Mean Squared Error (MSE)

Model <- lm(Measure~age+treatA+treatB, data=train_data)

summary(Model)

predict_lm <- predict(Model,test_data)

MSE.lm <- sum((predict_lm - test_data$Measure)^2)/nrow(test_data)

MSE.lm

#/Fuzzy Regression/

if(!require(fuzzyreg)) install.packages("fuzzyreg", dependencies = TRUE)

library(fuzzyreg)

require(limSolve)

require(quadprog)

f <-fuzzylm(Measure~age+treatA+treatB, data=train_data, method = "plrls", fuzzy.left.x = NULL,

 fuzzy.right.x = NULL, fuzzy.left.y = NULL, fuzzy.right.y = NULL)

coef(f)

**#/Checking for the missing values/**.

apply(bootdata, 2, function(x) sum(is.na(x)))

#/Scaling the data for normalization

max_data <- apply(bootdata, 2, max)

min_data <- apply(bootdata, 2, min)

data_scaled <- scale(bootdata,center = min_data, scale = max_data - min_data)

#/Randomly split the data into 70:30

index = sample(1:nrow(data_scaled),round(0.70*nrow(data_scaled)))

train <- as.data.frame(data_scaled[index,])

test <- as.data.frame(data_scaled[-index,])

#/Build the network, Create 3 hidden layers have 3 and 2 neurons respectfully

#Input layer = 2, Output layer = 1/

n = names(train)

f = as.formula(paste("Measure ~", paste(n[!n %in% "Measure"], collapse = " + ")))

nn = neuralnet(f,data=train,hidden=c(3,2),linear.output=T)

plot(nn)

options(warn=-1)

**#/30 percent of the available data to do this**:

predicted <- compute(nn,test[,1:3])

#/Compute Mean Squared Error of NN

MSE.net <- sum((test$Measure - predicted$net.result)^2)/nrow(test)

MSE.net

## Results

Multiple linear regression (MLR)

Table 3 presents the outcomes of multiple linear regression (MLR) applied to the training data. Significance was found in all model parameters concerning the effectiveness of treatment (p<0.0001). Additionally, the high adjusted R2 value (0.9503) indicates a strong model fit. Subsequently, 30% of the dataset was set aside for testing, yielding a mean square error (MSE lm) prediction of 9.975.

**Table 3:** MLR parameter estimates MLR - multiple linear regression

	Estimated	Std. error	t-value	p-values
Intercept	7.948	0.119	66.60	<0.001
Age	0.919	0.002	331.59	<0.001
Treatment A	25.075	0.103	241.92	<0.001
Treatment B	8.013	0.0911	87.93	<0.001

The equation derived from all independent variables is provided below:

Equation 7: effectiveness = 7.948 + 0.919 Age + 25.07 Treatment A+ 8.013 Treatment B

Fuzzy linear regression

The summary of the fuzzy linear regression (FLR) results generated using R-syntax is outlined in Table 4. This table includes the parameters derived from fuzzy linear regression, encompassing central, lower, and upper boundary values of the variables, as well as intercepts. Specifically, the central boundary values are utilized in preparing the fuzzy linear model, while the lower and upper boundary values are employed in formulating the equations for the lower and upper boundaries, respectively.

**Table 4:** Fuzzy linear regression parameter estimates by combining the bootstrap method

	Central tendency	Lower boundary	Upper boundary
Intercept	7.850	3.987	10.64
Age	0.913	0.913	0.913
Treatment A	25.32	25.32	26.17
Treatment B	8.604	8.604	9.34

Fuzzy linear regression is constructed using the central tendency values extracted from Table 3, while the lower and upper boundary fuzzy equations are formulated based on the corresponding columns in the table.

Equation 8: effectiveness = 7.850 + 0.913 (Age) + 25.32 (Treatment A) + 8.604 (Treatment B)

The lower boundary of the model support interval: 

Equation 9: effectiveness = 3.98 + 0.913 (Age) + 25.32 (Treatment A) + 8.604 (Treatment B)

The upper boundary for the model support interval:

Equation 10: effectiveness = 10.64 + 0.913 (Age) + 26.17 (Treatment A) + 9.345 (Treatment B)

Equations 9 and 10 accurately represent the full range of potential parameter values and predictions, providing a more comprehensive comprehension of the uncertainty linked to the fuzzy regression model. To assess the performance of the models constructed in equations 5 and 6, the effectiveness of treatment is evaluated using both MLR and FLR methodology. This evaluation involves calculating the predictive and actual values. Moreover, the absolute differences between actual and predicted values were calculated and small differences revealed the accuracy of the derived model. FLR demonstrates a marginally higher level of accuracy in comparison to MLR, as indicated by the average absolute difference (2.92 versus 2.99). Table 5 illustrates the comparison between the actual values of treatment effectiveness and the corresponding predicted values.

**Table 5:** Predicted effectiveness of treatment (measure) values from MLR and FLR regression models MLR - multiple linear regression; FLR - fuzzy linear regression

Original effectiveness values	Treatment type A	Treatment type B	Predicted values MLR	Absolute difference MLR	Predicted values FLR	Absolute difference FLR
56	1	0	52.317	3.683	52.343	3.657
41	0	1	37.188	3.812	37.453	3.547
40	0	1	43.621	3.621	43.844	3.844
28	0	0	25.409	2.591	25.197	2.803
55	1	0	58.75	3.75	58.734	3.734
25	0	0	29.085	4.085	28.849	3.849
46	0	1	46.378	0.378	46.583	0.583
71	0	0	69.521	1.479	69.021	1.979
Average				2.99		2.92

Multilayer Feedforward Neural Network for validation

**Figure 5:**
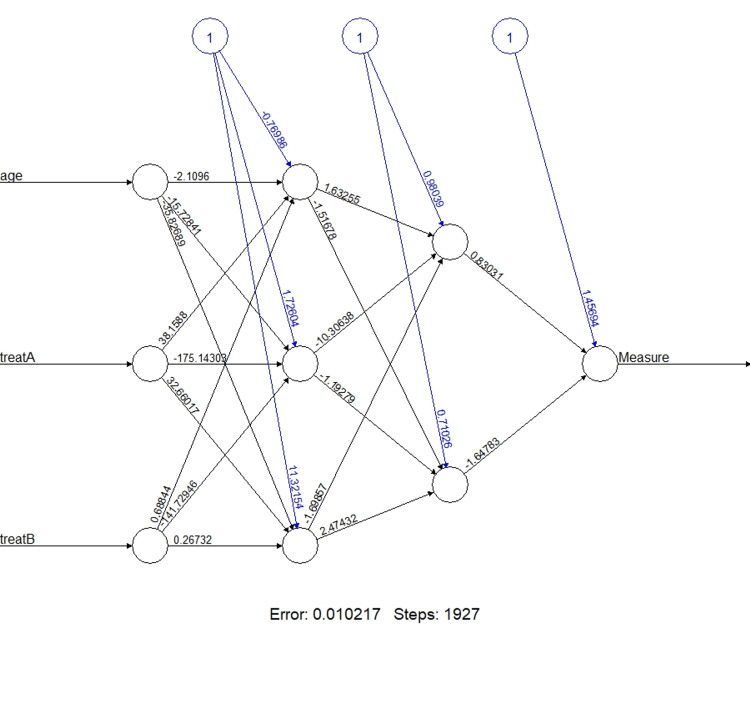
Architecture of the MLFFNN with three input variables, two hidden layers, and one output node (effectiveness of treatment) MLFFNN - Multilayer Feed Forward Neural Network

A Multilayer Feedforward Neural Network (MLFFNN) validated all variables included in the model. The MLFFNN's mean square error (MSE) is 0.362. The smallest MSE shows the best combination of the variables in the model and represents the shortest distance between the actual and predicted values of the model. Figure 5 shows the architectural diagram of MLFFNN. Table 6 shows the comparison of the predicted means square error (MSE) obtained from linear regression and MLFFNN. The low MSE value of MLFFNN indicated that the derived regression models are accurate, and model prediction will be more precise to be close to actual values. 

**Table 6:** Predicted mean square errors MLFFNN - Multilayer Feed Forward Neural Network; MSE - mean square error

Model	Error obtained
Linear regression (MSE lm)	9.975
MLFFNN (MSE net)	0.362

## Discussion

The proposed methodology for coordinating qualitative predictors within applied linear models represents a significant advancement in predictive modeling, particularly in the field of health science. This study presents a systematic approach to integrating qualitative predictors with quantitative variables in linear models. In this study, the transformation of qualitative variables was done using a dummy variable approach. Ahmad et al. (2022) predicted a model correlating waist circumference, HDL, and hypertension to normal, borderline, and high blood pressure triglycerides using a mixed dataset. The authors used dummy variables to add qualitative predictors to MLR and created a significant model indicating that HDL, waist, and hypertension impact triglycerides [15].

Similarly, after the transformation of qualitative predictors employed in the linear model to predict the effectiveness of treatment, the results indicated that the coefficients associated with age and treatment type were statistically significant (p-value <0.001). With an adjusted R2 value of 0.95, the regression model provides the greatest fit. However, linear regression performs well with crisp data [22]; if the underlying relationship does not adhere to the crisp function of the given form, linear model accuracy becomes questionable [23]. Therefore, fuzzy regression to efficiently account for the ambiguous relationship between the dependent and independent variables was applied. The estimated parameters for fuzzy regression parameters provide the fuzzy regression's central, lower, and upper boundaries for the fuzzy regression. The comparison of MLR and FLR models reveals that FLR exhibits a somewhat higher degree of precision compared to MLR (2.92 vs. 2.99).

Furthermore, the validity of the derived model was investigated through an MLFFNN. Güldoğan, in 2020, also used neural networks as a validation measure for the derived regression model's accuracy [24]. The implementation of the Multilayer Feedforward Neural Network (MLFFNN) via the backpropagation method was instrumental in enhancing prediction accuracy, as evidenced by the smaller mean squared error (MSE net) of 0.362. By utilizing techniques such as data screening, variable transformation, bootstrapping, MLR, FLR, and MLFFNN for validation, the methodology ensures the compatibility and robustness of the derived models.

Some limitations of the methodologies utilized in our study include the potential for multicollinearity arising from dummy variables, particularly with categorical variables featuring numerous levels. Another limitation is the computational complexity of using both fuzzy linear regression (FLR) and Multilayer Feedforward Neural Networks (MLFFNN). While fuzzy regression offers benefits in handling uncertainty and capturing nonlinear relationships, its interpretation can be more challenging compared to traditional linear regression due to the complexity of fuzzy logic principles and handling uncertainties. On the other hand, the computational complexity of MLFFNN primarily depends on the number of neurons in each layer, the number of layers, and the size of the training dataset. The model was constructed using a specific secondary dataset, so variations may arise when applying the methodology to different populations or settings.

## Conclusions

The proposed methodology demonstrated the precise and accurate estimation of predicted values using a secondary dataset. The hybrid approach enables a more robust estimation of model parameters by combining different techniques, resulting in improved model fitting, accuracy, and efficiency in parameter estimation efficiency. As a result, this hybrid method provides a viable solution for coordinating qualitative variables into linear models for high accuracy and strong prediction. In summary, the proposed technique, incorporating specific statistical tests, emerged as the most effective for modeling and prediction.
